# Integrating comprehensive care in the management of sickle cell disease patients in Nigeria

**DOI:** 10.1016/j.htct.2025.106091

**Published:** 2025-10-31

**Authors:** Efobi Chilota Chibuife, Nri-Ezedi Chisom Adaobi, Chilaka Ugochinyere Jenny, Okoye Helen Chioma, Anigbogu Ikechukwu Okwudili, Okwummuo Emeka Paul, Ogundeji Sunday Peter, Eze Onyinye Ezinne

**Affiliations:** a Nnamdi Azikiwe University, Department of Haematology and Blood Transfusion, Ituku-Ozalla Enugu, Nigeria; b Nnamdi Azikiwe University, Department of Paediatrics, Ituku-Ozalla Enugu, Nigeria; c Nnamdi Azikiwe University, Department of Haematology and Immunology, Nigeria; d University of Ibadan, Department of Haematology, Ituku-Ozalla Enugu, Nigeria; e ESUT Medical college, Department of Haematology and Blood Transfusion, Ituku-Ozalla Enugu, Nigeria

**Keywords:** Sickle cell disease, Comprehensive care, Haematologists, Paediatricians, Routine referrals

## Abstract

**Introduction:**

Comprehensive sickle cell care is a holistic, multidisciplinary approach spanning from birth to adulthood. It includes newborn screening, routine investigations, medications, specific therapies and structured referrals. It is recognised since the 1972 US Sickle Cell Control Act and reinforced by the American Society of Haematology initiatives. This study evaluates the adoption of these strategies by physicians in Nigeria.

**Aim:**

To examine the extent to which comprehensive care strategies are implemented in the management of sickle cell disease by adult and paediatric haematologists in Nigeria.

**Methodology:**

This cross-sectional study was conducted from September to November 2022 across six tertiary hospitals. An adapted and pretested primary care assessment tool was used to collect data on physician demographics and strategic components of comprehensive care. Descriptive statistics and chi-square tests were used to analyse the data.

**Results:**

A total of 157 doctors participated with most working in tertiary hospitals. Folic acid and proguanil hydrochloride were the most prescribed drugs; fewer than 50% used hydroxyurea. A complete blood count was the most requested investigation with 58% routinely scheduling investigations. Adult haematologists ordered more echocardiograms and paediatric haematologists requested more transcranial Dopplers. Adult haematologists referred more across specialities (p-value = 0.0001). All participants routinely counselled patients on clinic attendance, medication adherence and healthy lifestyle practices.

**Conclusion:**

Key components of comprehensive care are practised at varying levels by health professionals in Nigeria, mainly in urban/tertiary hospitals. To strengthen nationwide delivery of care, health policies should prioritise equitable workforce distribution and integration of additional services, like neonatal screening and emerging therapies.

## Introduction

Sickle cell anaemia is a monogenic disorder caused by a point mutation in the β-globin gene (*Glu6Val*) leading to the production of abnormal haemoglobin S. Under hypoxic conditions, haemoglobin S polymerizes, deforming red blood cells and triggering chronic haemolysis, inflammation, endothelial dysfunction, and activation of leukocytes and platelets. These mechanisms drive a wide range of complications affecting nearly every organ. Clinical heterogeneity is further influenced by co-inheritance of α-thalassaemia, foetal haemoglobin levels, hydroxyurea use, transfusion history, environmental factors, and use of emerging therapies [1,2].Nigeria bears the highest burden of sickle cell disease (SCD) globally, with an estimated 150,000 affected births annually and a general population prevalence of 2–3 % [3,4]. Comprehensive care, a multidisciplinary and preventive approach, has shown to improve survival and quality of life in SCD. Key elements include newborn screening, caregiver education, prophylactic antibiotics, vaccinations, malaria chemoprophylaxis, routine medications, clinical monitoring, and laboratory investigations. Effective pain management and timely treatment of vaso-occlusive crises are also essential [1,5,6].

Where available, other disease-modifying options such as the use of hydroxyurea, chronic transfusions, stem cell transplantation, and targeted molecular therapies are also used [5,7]. Hydroxyurea, increasingly recommended in comprehensive care, improves outcomes through multiple mechanisms, including increased foetal haemoglobin, reduced leukocyte and platelet activation and counts, improved red cell hydration, and reduced haemolysis [8].

Laboratory tests such as haemoglobin electrophoresis, complete blood counts (CBCs), liver and renal function tests, and reticulocyte counts support early diagnosis and monitoring.

Imaging tools, like transcranial Doppler and echocardiography, assist in stroke and pulmonary hypertension risk assessment, respectively. A multidisciplinary team including haematologists, paediatricians, nurses, psychologists, pain specialists, and social workers is essential for delivering coordinated care [5,6].

Despite the 2014 Nigerian national guidelines [9] addressing aspects of SCD care, including routine management and specialist referral [9], the absence of a structured care model and the lack of newborn screening severely limit the implementation of comprehensive care. Additionally, there is no unified national protocol, contributing to inconsistent practices among clinicians [10,11].

This study aims to assess the extent to which comprehensive care strategies are implemented in SCD management by adult and paediatric haematologists in Nigeria. It will evaluate the use of routine medications, diagnostic tools, and multidisciplinary services, with the goal of identifying gaps and informing future improvements in care delivery nationwide.

## Methodology

This multicenter cross-sectional study was conducted from September to November 2022across six tertiary institutions representing Nigeria’s six geopolitical zones. It assessed the extent to which adult and paediatric haematologists implemented comprehensive SCD care strategies in routine practice.

A modified and pre-validated version of the Primary Care Assessment Tool (PCAT) [12] was used. It was developed following focused group discussions by a selected group of locally based public health physicians and sickle cell care specialists. Using closed-ended questions, the tool collected data on the utilization of comprehensive services like routine clinical assessments, organ function assessments, use of routine and disease-modifying treatments, access to multidisciplinary care and physician demographics including years of practice, location, speciality, and cadre.

Content validity was established through expert review by three adult and three paediatric haematologists. The modified PCAT tool was deployed through Google Forms. An informed consent statement outlining the study title, purpose, benefits, risks and voluntary nature of participation was presented at the beginning. Completion of the form is possible within ten minutes, and consent was indicated by checking the “YES” option. In each participating centre, a designated focal person, a local SCD specialist, disseminated the tool and coordinated data collection. These focal persons, familiar within their communities, enhanced participation by sending reminders and making phone calls to limit attrition. They received no financial incentives.

Given an estimated number of 10 to 20 adult haematology department consultants and residents, and 8 to 15 paediatric haematology department consultants and residents per centre, the calculated estimated total population n was derived as shown below.

The average number of sickle cell care doctors per centre

$\frac{8+15}{2}$ = 11.5 for paediatric haematology

$\frac{10+20}{2}$ = 15 for adult haematology

The total number of specialists per centre: 11.5 + 15 = 26.5.

The total number of specialists across six centres: 26.5 × 6 = 159.

Estimated total population: *n* = 159

With a 95 % confidence level, a 5 % margin of error, and a p-value = 0.05, and using the Cochran formula with a finite population correction, the total estimated sample size was 113 participants.

To ensure an equitable centre-wide representative participation, the sample was proportionally distributed based on the number of eligible participants per centre. The recruitment targets illustrated in Table 1 present the estimated staff strength per centre and the corresponding sample size.

**Table 1:** Recruitment targets.

Centre	Estimated number from paediatric haematology	Estimated number from adult haematology	Total number of the centre	Allocated sample size
A	8	10	18	13
B	10	15	25	18
C	12	20	32	23
D	15	18	33	24
E	11	14	25	18
F	12	14	26	17
Total			159	113

Sample size per centre = (Centre size divided by 159) x 113

**Figure. 1:**
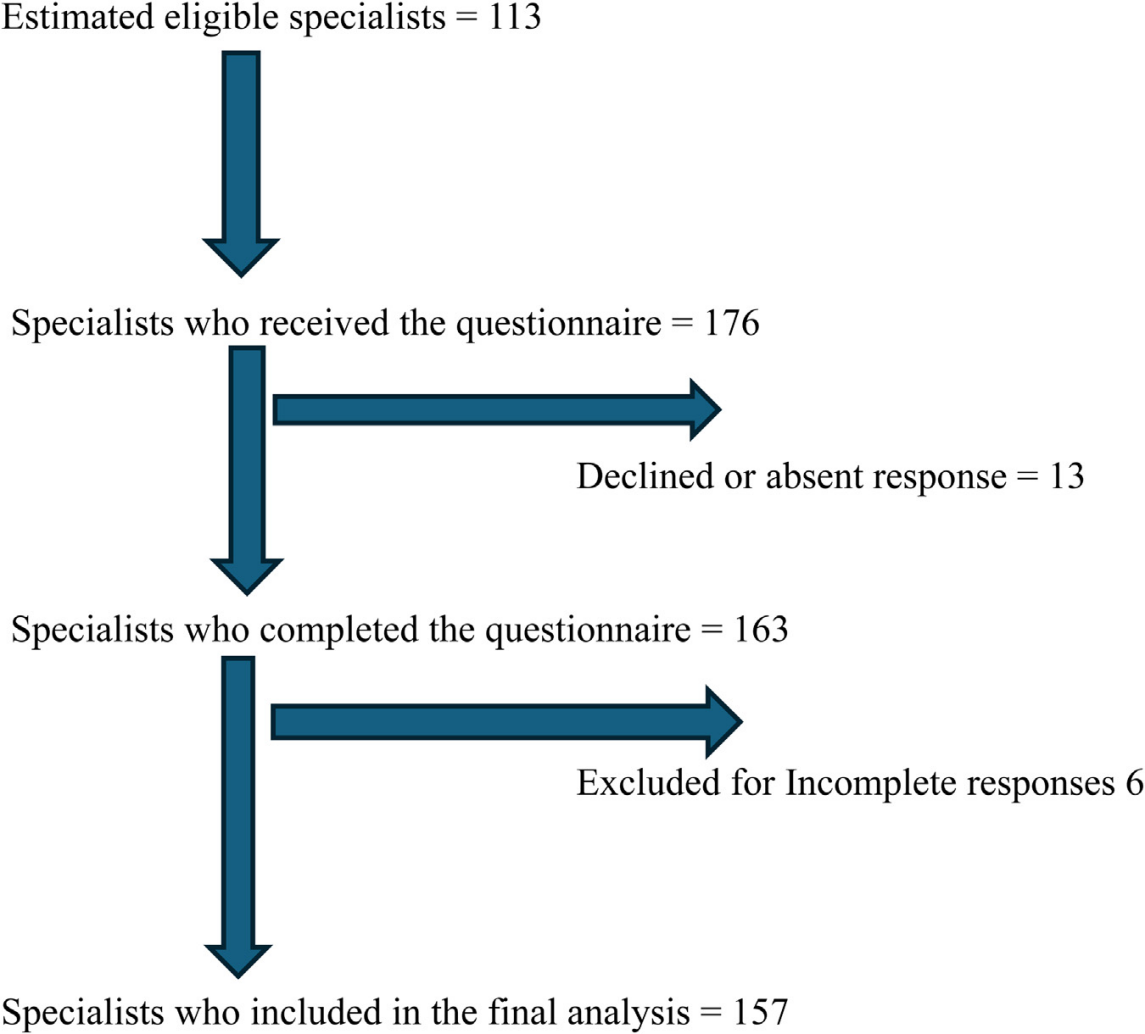
The flow of participant inclusion from eligibility to data analysis. The response rate = The total number of completed questionnaires ÷ by the number who received the questionnaire × 100 = approximately 93 %.

Mixed sampling was used. Institutions with the largest staff strength per region were purposively selected. Subsequently, a census approach was used to recruit all eligible specialists within the centres. Sampling was proportionally stratified based on staff strength to ensure balanced representation across the regions. A total of 157 duly answered questionnaires were returned during the study thus the response rate was 93 % (Figure 1).

The University of Nigeria Teaching Hospital's Ethics Review Board granted ethical approval.

### Inclusion and exclusion criteria

Adult haematology physicians and paediatric haematology physicians were included in this study. The following were the exclusion criteria: physicians in other clinical specialities who do not routinely manage patients with SCD; physicians who did not complete the online questionnaire; and physicians who did not give consent

### Data analysis

Descriptive statistics were done using the STATA computer program (version 16.1). Sociodemographic and study-related characteristics, such as the use of routine medications, frequency and type of routine investigations, and physician referral habits and triggers for such referrals, were analysed and presented as frequencies, charts and in text. The chi-square test was used to assess the association between categorical variables.

## Results

### Recruitment summary

A total of 176 physicians received the questionnaire, 163 completed it, and 157 were included in the final data analysis after excluding six incomplete responses. Thirteen participants declined to participate. The participant flow chart is shown in Figure 1

### Physician demographics

A total of 157 doctors participated in the study, comprising 54.1 % males and 45.9 % females. The mean age of respondents was 42 years, with an age range of 36–48 years. Among the participants, 85 were from the adult haematology departments and 72 from the paediatric haematology departments.

In the adult haematology group, consultants comprised the majority of participants (*n* = 60). Within the paediatric haematology group, 44.4 % were consultants, while the remaining 55.6 % were other cadres of doctors.

Geographically, 56 % of respondents were based in southern Nigeria, while 44 % were based in northern Nigeria. Within the southern region, South-Eastern Nigeria contributed the highest proportion of participants (28 %), while in the northern region, North-Western Nigeria had the largest representation (20.4 %).

Regarding the type of health facility, 79 % of participants were affiliated with tertiary hospitals, 3.8 % with private hospitals, and 0.6 % with primary healthcare centres. In terms of professional experience, approximately 50 % of respondents reported 11–15 years of clinical practice, while 25 % had 16–20 years of experience.

**Figure. 2:**
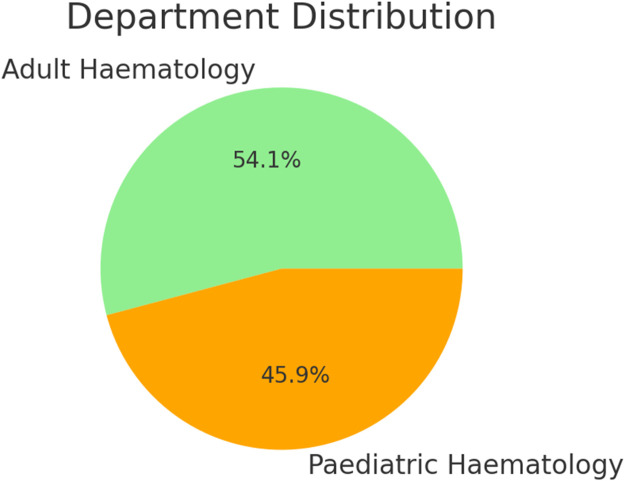
Physician demographics.

**Figure. 3:**
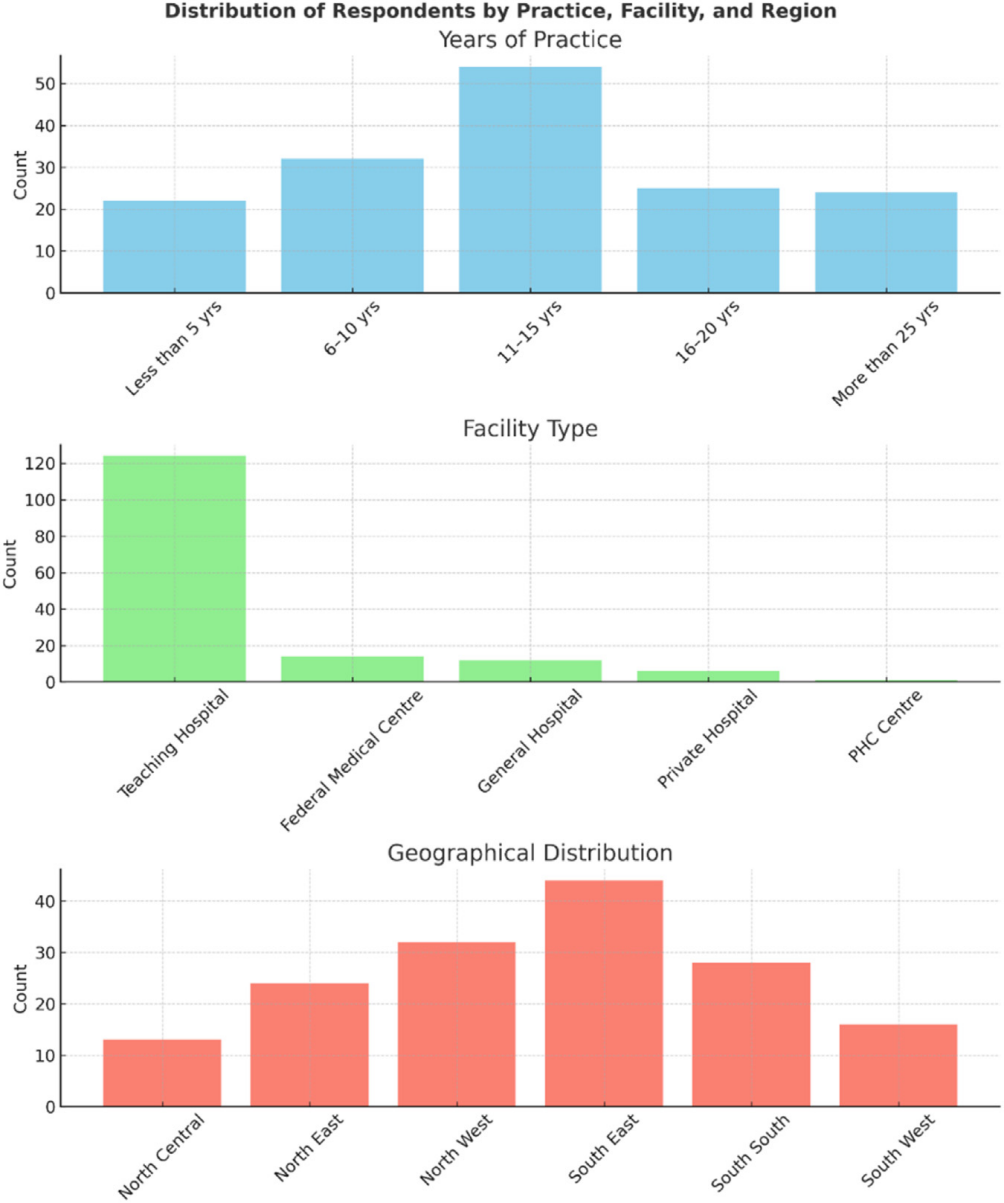
Physician demographics: Facility type, years of practice and geographical region.

Figure 2 shows the percentage distribution of physicians from adult haematology and paediatric haematology. Figure 3 is the distribution of respondents by facility type, years of practice and geographical region.

### Use of both routine and disease-modifying medications

#### Prescription of routine medications

The pattern of routine drug prescriptions among adult and paediatric haematologists is illustrated in Table 2. Among adult haematologists, the most frequently prescribed medications, in descending order, were folic acid, proguanil hydrochloride, vitamin C, hydroxyurea, vitamin B complex, a fixed multivitamin preparation, Omega-3 fatty acids, penicillin, fixed multivitamin-amino acid supplements, and ferrous fumarate. In comparison, paediatric haematologists mainly prescribed folic acid, proguanil hydrochloride, penicillin, hydroxyurea, vitamin C, fixed multivitamin preparations, vitamin B complex, fixed multivitamin-amino acid supplements, ferrous fumarate, and omega-3 fatty acids, in that order.

**Table 2:** Use of routine and disease-modifying drugs.

Routine Drugs	Adult Haematology n ( %)	Paediatric Haematology n ( %)
Folic acid	85.0 (100.0)	69.0 (95.8)
Proguanil hydrochloride	78.0 (91.8)	62.0 (86.1)
Vitamin C	47.0 (55.3)	26.0 (36.1)
Hydroxyurea	42.0 (49.4)	34.0 (47.2)
Vitamin B complex	33.0 (38.8)	13.0 (18.1)
Multivitamins	20.0 (23.5)	16.0 (22.2)
Omega 3 fatty acids	16.0 (18.8)	3.0 (4.2)
Penicillin	8.0 (9.4)	42.0 (58.3)
Fixed multivitamin-amino acids supplements	3.0 (3.5)	13.0 (18.1)
Ferrous fumarate	2.0 (2.4)	4.0 (5.6)

Folic acid and proguanil hydrochloride were the two most prescribed medications across both groups. However, fewer than 50 % of physicians in either group reportedly routinely prescribing hydroxyurea. Notably, penicillin was prescribed significantly more often by paediatric haematologists compared to their adult counterparts.

#### Organ function assessments

As shown in Table 3, the CBC was the most frequently requested investigation by both groups. In contrast, fundoscopy and hepatitis screening were the least commonly requested. A Chi-squared analysis revealed that adult haematologists requested echocardiographies significantly more frequently than paediatric haematologists, whereas paediatric haematologists were significantly more likely to request transcranial Doppler (TCD) assessments.

**Table 3:** Organ function assessments.

Routine Tests	Adult Haematology n ( %)	Paediatric Haematology n ( %)	P-value
Complete Blood Count	82.0 (96.5)	57.0 (79.2)	
Urinalysis	54.0 (63.5)	24.0 (33.3)	
Kidney Function Test	45.0 (52.9)	28.0 (38.9)	
Liver Function Test	32.0 (37.6)	17.0 (23.6)	
Transcranial Doppler Ultrasound	16.0 (18.8)	27.0 (37.5)	
Echocardiography	13.0 (15.3)	2.0 (2.8)	<0.0001
Chest X-ray	1.0 (1.2)	0.0 (0.0)	
Abdominal Ultrasound	0.0 (0.0)	0.0 (0.0)	
Packed cell Volume (PCV)	1.0 (1.2)	8.0 (11.1)	
Thick film for malaria Parasite	1.0 (1.2)	0.0 (0.0)	
Peripheral Blood Film	1.0 (1.2)	0.0 (0.0)	
Fundoscopy	0.0 (0.0)	1.0 (1.4)	
Hepatitis Screen	0.0 (0.0)	1.0 (1.4)	
Routine request not performed	2.0 (2.4)	7.0 (9.7)	

#### Frequency of organ function assessments

Table 4 presents the intervals at which routine organ function investigations were requested. Only 20.4 % of physicians ordered tests monthly, 28.0 % did so quarterly, and 10.8 % did so yearly. A notable 35.5 % of respondents reported no consistent schedule for requesting routine investigations, while 6.3 % did not order routine investigations.

**Table 4:** Frequency of organ function assessments.

	Total (*n* = 157)	Adult Haematology (*n* = 85)	Paediatric Haematology (*n* = 72)	p-value
Not routinely requested	10 (6.3 %)	2 (2.4 %)	8 (11.1 %)	
Not consistent	54 (34.4 %)	25 (29.4 %)	29 (40.3 %)	
Monthly	32 (20.4 %)	25 (29.4 %)	7 (9.7 %)	
Quarterly	44 (28.0 %)	25 (29.4 %)	19 (26.3 %)	0.008
Yearly	17 (10.8 %)	8 (9.4 %)	9 (12.5 %)	

#### Access to multidisciplinary care

The referral patterns of participating physicians are summarized in Table 5. Clinical need was the predominant reason for referral among both groups. Across all other specialties, adult haematologists referred patients significantly more frequently than paediatric haematologists (Chi-squared test: p-value = 0.0001). The most frequently referred specialties by adult haematologists, in descending order, included obstetrics and gynaecology, ophthalmology, psychiatry, otorhinolaryngology, and dentistry. Interestingly, both adult and paediatric haematologists rarely referred patients to urologists or to fellow haematologists. Routine referrals were not practiced by 62.5 % and 37.6 % of paediatric haematologists and adult haematologists, respectively.

**Table 5:** Access to multidisciplinary care.

Referred to Speciality	Haematology	Paediatric Haematology	p-value
Referrals not routine	32.0 (37.6)	45.0 (62.5)	
Obstetrician	47.0 (55.3)	12.0 (16.7)	
Ophthalmologists	33.0 (38.8)	12.0 (16.7)	
Psychiatrist	20.0 (23.5)	5.0 (6.9)	
ENT Surgeon	14.0 (16.5)	5.0 (6.9)	
Dentists	12.0 (14.1)	0.0 (0.0)	<0.0001
Nephrologist	3.0 (3.5)	0.0 (0.0)	
Orthopaedic surgeon	3.0 (3.5)	1.0 (1.4)	
Pulmonologist	1.0 (1.2)	0.0 (0.0)	
Neurologist	0.0 (0.0)	1.0 (1.4)	
Haematologist	0.0 (0.0)	3.0 (4.2)	
Paediatrician	0.0 (0.0)	1.0 (1.4)	
Urologist	1.0 (1.2)	0.0 (0.0)	

#### Patient education and counselling

**Figure. 4:**
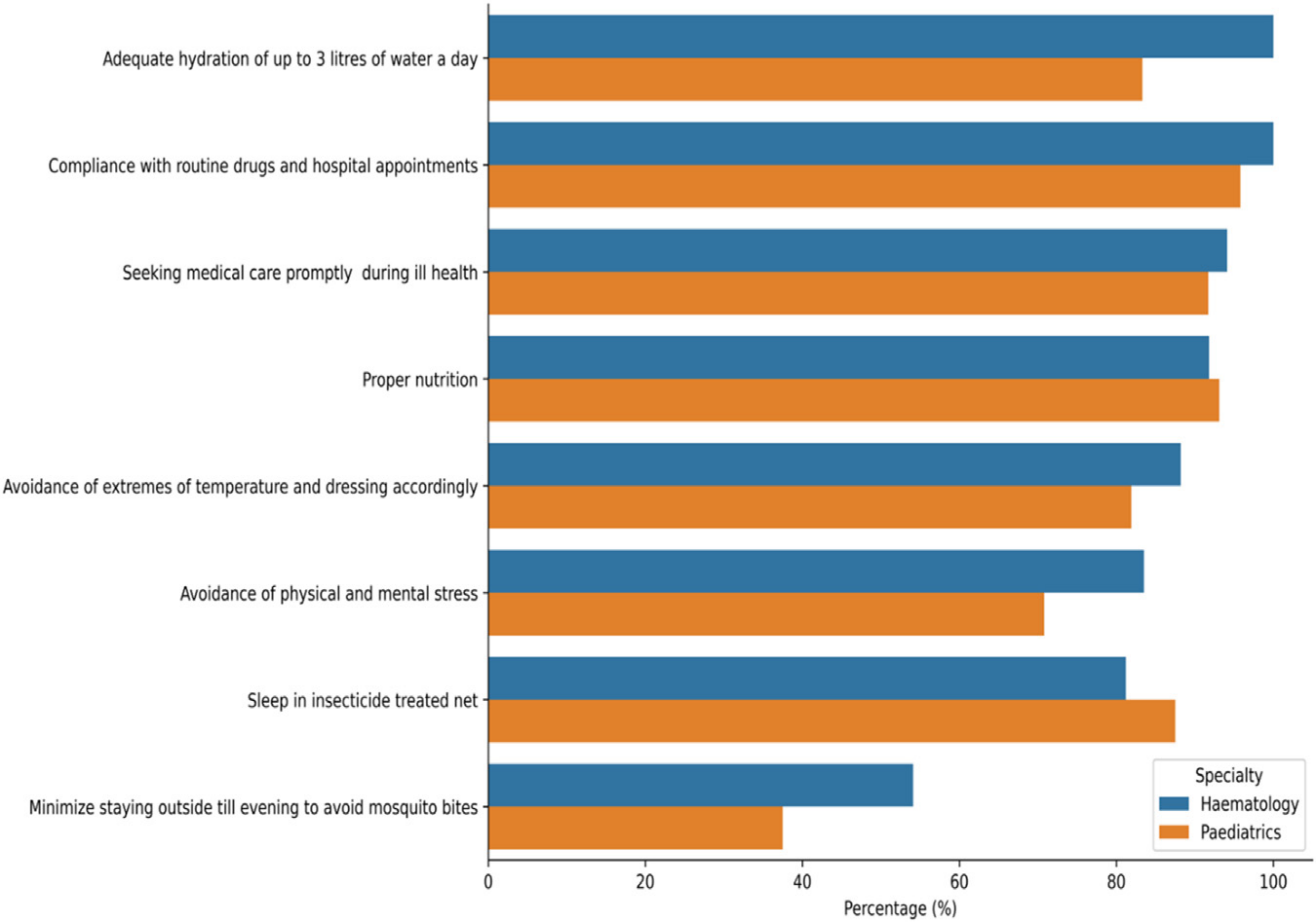
Patient education and counselling.

As shown in Figure 4, all participating physicians routinely counselled patients on regular clinic attendance, adherence to prescribed medications, proper nutrition, healthy habits, malaria prevention strategies and other lifestyle modifications.

## Discussion

Comprehensive care remains a cornerstone of SCD management as endorsed by the World Health Organization, the American Society of Hematology, and the Nigerian government. It encompasses a wide range of clinical interventions, from scheduled services such as neonatal screening, routine disease monitoring to prophylactic and disease-modifying therapies, all aimed at reducing morbidity and improving quality of life [9,13,14].

This study revealed that 90.4 % of respondents were SCD specialists from either adult haematology or paediatric haematology, primarily working in tertiary centres. Over 85 % had more than six years of clinical experience, aligning with evidence that specialist-led care improves outcomes in SCD [15,16]. However, about 95 % were in urban centres, reflecting the ongoing maldistribution of healthcare professionals in sub-Saharan Africa, which continues to disadvantage rural populations and hinder equitable access to care [6,17].

Contrary to reports by Kanter et al. [6] that highlight more paediatric haematologists in SCD care, this study found a slight predominance of adult haematologists. This may reflect institutional variability or gaps in our national specialist registries and is thus not representative of the actual number in the country. More critically is the broader challenge of a limited workforce for SCD and other haematological disorders in Nigeria and Sub-Saharan Africa [3,8,18].

All participating specialists routinely prescribed core medications, demonstrating that they are integral to comprehensive care [3,8]. Folic acid, antimalarials (e.g., proguanil), and penicillin were most used, while hydroxyurea, despite its proven disease-modifying potential, was underutilized. This discrepancy likely stems from issues of cost, availability, limited treatment guidelines, safety concerns regarding use in pregnant and lactating mothers and as a possible cause of infertility [19,20].

The preference for penicillin among paediatricians aligns with evidence from the PROPS study, which demonstrated its efficacy in preventing life-threatening infections in children with SCD [21]. Notwithstanding, the 58 % uptake recorded by the paediatric haematologists, the rate of penicillin prophylaxis is deemed low, though this is in keeping with other studies [22]. Contributing factors include cost, limited availability of paediatric formulations, poor adherence due to SCD chronicity, sociocultural barriers, provider reluctance linked to the absence of national guidelines, the inclusion of vaccines for encapsulated organisms in routine immunization which has reduced the emphasis on prophylaxis and *Salmonella typhi* not *Streptococcus pneumonia* having been the most common isolate in a Nigerian study [23, 24, 25].

Folic acid supplementation remains a standard component of care despite ongoing debate about its clinical benefits. A Cochrane review suggests that aside from an increase in serum folate levels, it has limited impact on anaemia severity [26].

Routine folic acid use is usually justified by a theoretical need to prevent deficiency from increased folate turnover due to chronic haemolysis. Additionally, hypermetabolism in haemoglobin SS, elevated interleukin-6 levels (which may suppress appetite), and increased resting energy expenditure contribute to micro- and macro-nutrient deficiencies, underscoring the need for nutritional replacement in sickle cell anaemia management [26,27].

Vitamin B complex, other multivitamin preparations, and anti-inflammatory agents like omega-3 fatty acids are not as regularly prescribed because they are primarily used as supplements and, unlike the others, do not directly influence the pathophysiological mechanisms and complications of SCD.

Regarding the use of vitamin C, it is postulated to be a potent antioxidant that shields red cell membranes from damage by reducing the generation of reactive oxygen species [28]. In addition, vitamin C also promotes the absorption and metabolism of iron with improvements in specific red cell indices [29]. Iron supplementation is the least prescribed and is usually for coexisting iron deficiency anaemia.

Routine laboratory monitoring is a key pillar of comprehensive care. In this study, most specialists conducted regular tests, including CBCs, urinalysis, and renal function assessments.

Adult haematologists prioritized echocardiography to detect complications like pulmonary hypertension common in older SCD patients [1,3]. In contrast, paediatricians more frequently requested TCD ultrasound to screen for stroke risk, consistent with its established utility in children with SCD. This, in part, is due to established guidelines recommending annual screening for children with SCD, which the ASH guidelines, as well as the STOP and SPRING trials, have significantly influenced. TCD, as a standard of care in paediatric clinics, is supported by public health initiatives and advocacy primarily targeting children with sickle cell anaemia. In contrast, adult SCD care has limited TCD validation studies and lacks structured protocols. These factors collectively drive the increased TCD requests in Paediatric settings [30,31].

Referrals to other specialities were frequently prompted by specific clinical concerns such as stroke, priapism, leg ulcers, or mental health issues.

This multidisciplinary approach includes psychiatrists, surgeons, nephrologists, gastroenterologists, neurologists, social workers, nutritionists, pain specialists etc. It reflects best practice models and is central to holistic SCD care. Counselling also featured prominently, highlighting the psychosocial burdens of SCD and the importance of integrated mental health support. Each group consistently emphasized health-seeking and other well-being habits. Of key importance is malaria prevention because malaria infection is endemic in the population

While this study confirms a growing commitment to comprehensive, specialist-led SCD care in Nigeria, significant gaps remain. Chief among these is the limited rural coverage of specialist services, inconsistent access to hydroxyurea and diagnostics, and the absence of standardized care protocols. These deficiencies contribute to fragmented care delivery and suboptimal outcomes, particularly outside tertiary centres. Furthermore, even within tertiary facilities, the lack of standardized care protocols and harmonized clinical guidelines has led to inadequacies and considerable variability in the application of comprehensive care strategies by physicians. Addressing these gaps will require targeted national surveys and the constitution of expert panels to develop standardized and universally accepted comprehensive care protocols. Additionally, greater advocacy is needed to raise awareness of these deficiencies and to address specific practice gaps, such as the reduced use of TCD screening in adult haematology care. This should be supported by further validation studies focused on the utility of TCD in adult SCD populations.

This study did not capture essential aspects of comprehensive care, such as newborn screening, vaccinations, and the use of emerging therapies, including gene and targeted therapies, in the management of SCD. This is due to the widespread unavailability of these services, which is a consequence of financial, infrastructure, and socioeconomic challenges, resulting in a weakened health service framework. The small sample size, purposive site selection a cross-sectional design and the answers of the surveys not validated against the medical records may create bias and limit the generalizability of study findings

## Conclusion

This study underscores the importance of structured, comprehensive care in SCD management and affirms the critical role of haematology specialists in delivering this care. Although implementation is significant in urban tertiary centres, systemic barriers, including workforce shortages, urban concentration of expertise, and inconsistent access to medications, impede equitable care delivery nationwide. Addressing these challenges requires strategic policy actions, including decentralisation of services, and the expansion of care services and the SCD workforce, with improved funding for essential medicines and enhanced diagnostics.

## Author contributions

Efobi CC participated in conceptualization, manuscript drafting, study design, data collection, interpretation, Statistical analysis, and manuscript reviews.

Nri-Ezedi CA participated in conceptualization, manuscript drafting, study design, data collection, interpretation, Statistical analysis, and manuscript reviews.

Chilaka UJ participated in conceptualization, manuscript drafting and study design, data collection, interpretation and manuscript reviews.

Okoye HC participated in conceptualization, manuscript drafting and study design, data collection, interpretation and manuscript reviews.

Anigbogu IO participated in conceptualization, manuscript drafting and study design, data collection, interpretation and manuscript reviews.

Okwummuo EP participated in conceptualization, manuscript drafting and study design, data collection, interpretation and manuscript reviews.

Ogundeji P S participated in conceptualization, manuscript drafting, study design, data collection, interpretation, and manuscript reviews.

All authors participated in the final approval of the manuscript.

## Data availability

Data sets and other study documents are appropriately secured and will be available upon request.

## Ethics statement

The University of Nigeria Teaching Hospital's Ethics Review Board granted ethical approval.

## Source of funding

This research did not receive any specific grants from funding agencies in the public, commercial or not for profit sectors.

## Conflicts of interest

The authors declare there is no conflict of interest in undertaking this study.
